# Heteroplasmy in action: tracking mtDNA segregation dynamics

**DOI:** 10.1038/s44318-024-00226-x

**Published:** 2024-09-10

**Authors:** Nitish Dua, Anjana Badrinarayanan

**Affiliations:** 1 The Scripps Research Institute, La Jolla, CA USA; 2 National Centre for Biological Sciences (TIFR), Bengaluru, Karnataka 560065 India

**Keywords:** Organelles

## Abstract

A powerful new approach for monitoring heteroplasmic yeast populations in real-time has revealed insights into the forces underlying mtDNA variant segregation.

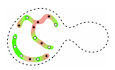

## Teaser

Difficulties in tracking the dynamics of heteroplasmy, a state when a single cell contains distinct mtDNA genotypes, have left our understanding of factors influencing it incomplete. New work now provides a powerful approach for monitoring heteroplasmic yeast populations in real-time, and reveals insights into the forces underlying mtDNA variant segregation.

Heteroplasmy arises when a single cell contains distinct mtDNA genotypes. The degree of heteroplasmy varies depending on the rates of mutation accumulation across the lifetime of an organism, and this can have important consequences on mitochondrial function (Wallace and Chalkia, 2013). Tracking heteroplasmic dynamics is challenging, and hence we do not yet have a complete understanding of the factors influencing the levels of heteroplasmy in the population. In a new study, Roussou et al develop a powerful approach that enables them to follow heteroplasmic yeast populations in real-time, at single-cell resolution across several generations of growth (Roussou et al, 2024). The authors leverage fluorescent markers indicative of mtDNA variants and visualize their segregation dynamics using microfluidics and automated cell tracking methods. They complement this quantitative approach with mathematical modeling to reveal asymmetric segregation, as well as mitochondrial fission and fusion dynamics, as key forces that drive mtDNA variant segregation.

Mitochondria contain multiple small genomes, which encode for proteins involved in oxidative phosphorylation. These genomes are indispensable for mitochondrial function. Similar to the nuclear genome, mtDNA faces stress due to genotoxic agents and replication errors resulting in the accumulation of mutations on individual mtDNA molecules. This leads to a condition called heteroplasmy, where a single cell contains a mix of mutant and wild-type mtDNA copies (Stewart and Chinnery, 2015). The effect of these mutations on mitochondrial function is dependent on the level of heteroplasmy and the nature of the mutation. In healthy cells, heteroplasmy can be relatively benign, especially in the case of neutral or mildly deleterious mutations (that can be buffered by the wild-type copies). However, if the mutation load crosses a certain threshold, typically around 60–90% depending on the specific mutation and cell type, mitochondrial function can be severely compromised (Stewart and Chinnery, 2015).

What factors contribute to the development of heteroplasmy? The variability in heteroplasmy has been predominantly attributed to relaxed replication and vegetative segregation of mtDNA (Stewart and Chinnery, 2015). During relaxed mtDNA replication, which occurs independent of the nuclear genome replication, both wild-type and mutant mtDNA copies can expand in individual cells in the population. On the other hand, asymmetric partitioning of these wild-type and mutant mtDNA copies during division can give rise to different levels of heteroplasmy between mother and daughter cells, and the gradual dominance of one mtDNA variant over others (homoplasmy). Some pathways can tilt the balance towards homoplasmy in the population. For example, bottlenecking in germline cells can have drastic effects on levels of heteroplasmy, albeit this can work in favor of either wild-type or mutant mtDNA copies (Wallace and Chalkia, 2013). There is also evidence in *Drosophila*, showing selectivity in replication of wild-type mtDNA during germline cell development (Hill et al, 2014). Contrary to this, expansion of mutant mtDNA over wild-type has also been observed in *C. elegans* (Yang et al, 2022). In addition, theoretical models have proposed that the distribution of mtDNA throughout the mitochondrial network, and its partitioning during cell division, can influence the variability of heteroplasmy within the population.

However, direct observation of mtDNA variant dynamics in living cells has been challenging. A major hurdle has been the inability to track mtDNA molecules at single cell level, and over the long timescales over which heteroplasmic dynamics are expected to play out. Most early studies have relied on static measurements of heteroplasmy levels, which provides limited temporal insights into mtDNA variant segregation and the factors influencing these processes. More recently, sequencing techniques have provided population-level analysis of changes in heteroplasmy, but this averages out cell-to-cell variability. Some exciting new technological advancements are breaking the barriers to reveal new insights on heteroplasmy. For example, Kotrys et al recently described a method leveraging single-cell combinatorial indexing (SCI) in conjunction with Split-seq for the assessment of heteroplasmic mtDNA sequences at single-cell level (Kotrys et al, 2024). Li et al also devised an imaging-based method to visualize mutant and wild-type mtDNA using a Cas12-based sensor, which can be applied to assess levels of mtDNA heterogeneity (Li et al, 2023).

**Figure 1:**
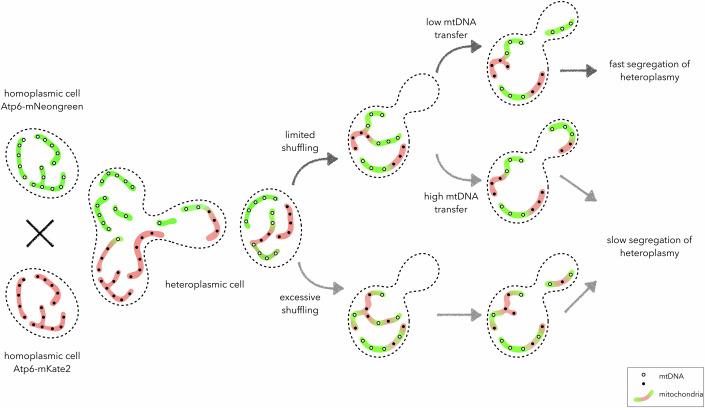
In the new system for tracking heteroplasmy in real-time, budding yeast cells homoplasmic for mtDNA variants expressing either ATP6-mNeongreen or ATP6-mKate2 are used to generate heteroplasmic diploids. Mitochondrial fission and fusion rates influence the shuffling of mtDNA variants in these diploids. Together with this, asymmetric mtDNA transfer to the daughter cells determines the rate of heteroplasmic mtDNA segregation. See text for details.

Roussou et al (2024) make a significant breakthrough by developing a sensitive assay to track heteroplasmy in single cells. The authors utilize their previously described approach (Jakubke et al, 2021) to make budding yeast strains expressing mitochondrially encoded Atp6-mNeongreen and Atp6-mKate2. They use these two homoplasmic strains to generate a diploid zygote containing a mix of both mtDNA molecules (Fig. 1). Using fluorescence as an indicator for the presence of these mtDNA molecules, the authors track, in microfluidic devices, the segregation of heteroplasmic mtDNA in single cells.

It is striking to note that a rapid transition toward homoplasmy is observed within these yeast populations. The authors demonstrate that cells starting with a near-equal mix of two mtDNA variants can become dominated by one variant within just a few hours, suggesting that mtDNA segregation is unlikely to be purely stochastic. This segregation occurs with no preference for a specific mtDNA genotype, since both the copies are functional. Combining mathematical modeling with experimentally generated data, the authors show that the amount of mtDNA transferred to the daughter cells influences the rates at which cells become homoplasmic. Their results estimate that ~11 mtDNA nucleoids are transferred to daughter cells, which then replicate further in the bud. It is tempting to suggest that this resembles a genetic bottleneck observed in germline cells.

A major contributing factor to the segregation dynamics are the rates of mitochondrial fission and fusion. Previous work has shown that mitochondrial division plays a central role in determining the distribution of mtDNA within the mitochondrial network (Lewis et al, 2016). Here the authors demonstrate how these dynamics influence heteroplasmy levels. They show that limited fission and fusion is required for the segregation of mtDNA variants, with reduced fusion-fission cycles leading to faster segregation of mtDNA variants, driving the population toward homoplasmy.

Using this system, the authors proceed to ask if the segregation dynamics change when one of the mtDNA copies contains a mutation. In this case they find that mtDNA segregation works in favor of the wild-type mtDNA molecules. This purifying selection supports the prevailing model of selective segregation of “fitter” mitochondria into the daughter cells (Vevea et al, 2014), in this case reflecting mtDNA fitness. Such selective segregation of mitochondria has been observed under conditions of mtDNA damage and even in adult human stem cells (Dua et al, 2022; Katajisto et al, 2015). Interestingly, in mammalian cells heteroplasmy persists when synonymous mutations are present (Kotrys et al, 2024), while asymmetric partitioning of mtDNA in budding yeast occurs irrespective of impact of the mtDNA mutation on fitness.

The study by Roussou et al (2024) has important implications for understanding the mechanisms of purifying selection against mtDNA mutations. Several mutations on mtDNA have been found to be associated with human diseases. These mutations can occur in somatic cells and manifest as late-onset disorders or, if inherited, can present as pathologies since birth (Stewart and Chinnery, 2015). Levels of heteroplasmy have also been found to increase with age in humans, and vary drastically in a tissue-specific manner (Sanchez-Contreras et al, 2023). Although pathogenic heteroplasmy is observed in a large population of adult humans, and its relation to ageing and disease pathogenesis is well established, our understanding of the dynamics of heteroplasmy and what factors give rise to the variability amongst tissue types remains incomplete.

The real-time data generated by Roussou et al (2024) shows that the physical partitioning of mtDNA during cell division is not random but is instead influenced by the dynamics of mitochondrial fusion and fission. With the ability to track mtDNA heteroplasmy over multiple generations, the authors pave the way for identifying the molecular players that facilitate this purifying selection of mtDNA. For example, it would be interesting to know which, and how, nuclear-encoded factors involved in cristae remodeling, organelle contacts and the cytoskeleton affect mtDNA variant segregation. Since some mtDNA mutations are relatively more penetrant, such mutants could also be studied in the present system to understand the effects of different mutations on heteroplasmy dynamics. Indeed, this could provide a framework for exploring similar processes in mammalian cells, where mtDNA mutations are linked to a wide range of diseases and aging-related decline in mitochondrial function.
